# Development of Eosinophilic Granulomatosis With Polyangiitis Despite Anti–Interleukin‐5 Receptor Therapy: The First Case of Bilateral Central Retinal Artery Occlusion During Benralizumab Treatment

**DOI:** 10.1002/acr2.70073

**Published:** 2025-06-03

**Authors:** Elena Treppo, Lucia Finocchio, Benedetta Fazzi, Maria Pillon, Luca Quartuccio

**Affiliations:** ^1^ Division of Rheumatology, Department of Medicine University of Udine, Azienda Sanitaria Universitaria del Friuli Centrale Udine Italy; ^2^ Department of Ophthalmology Azienda Sanitaria Universitaria del Friuli Centrale Udine Italy

## Abstract

Here, we describe a rare presentation of eosinophilic granulomatosis with polyangiitis (EGPA) under benralizumab therapy manifesting as bilateral central retinal artery occlusion (CRAO). The patient, a 61‐year‐old man with chronic eosinophilic rhinosinusitis and severe asthma, experienced sudden bilateral visual loss and transient amaurosis. Ophthalmologic evaluations, including a fundus examination and optical coherence tomography, confirmed CRAO, and laboratory test results revealed elevated markers of inflammation and positive antimyeloperoxidase antibodies in the context of normal eosinophil counts. Intensive immunosuppressive therapy led to resolution of systemic inflammation, although significant visual impairment persisted. These findings underscore the potential limitations of anti–interleukin‐5 receptor therapy in preventing vasculitic complications in EGPA.

## Introduction

Eosinophilic granulomatosis with polyangiitis (EGPA) is a rare vasculitis affecting small to medium vessels characterized by a clinical spectrum ranging from predominantly eosinophilic inflammation to vasculitic lesions and is sometimes associated with antineutrophil cytoplasmic antibodies (ANCAs). Ocular involvement in EGPA is notably uncommon, and central retinal artery occlusion (CRAO) represents an exceedingly rare manifestation.^1^ With the recent adoption of benralizumab, an anti–interleukin‐5 (anti–IL‐5) receptor therapy, for severe eosinophilic asthma, its effect on preventing vasculitic complications remains uncertain. The first reported case of EGPA with bilateral CRAO arising during benralizumab therapy is presented here.

## Case report

The patient, a 61‐year‐old man with a history of chronic eosinophilic rhinosinusitis previously treated with functional endoscopic sinus surgery and severe eosinophilic asthma managed with benralizumab at 30 mg every eight weeks (initiated in 2022), was referred to the emergency department in June 2024 for sudden, painless bilateral vision loss. In the weeks preceding admission, he experienced recurrent episodes of transient, bilateral amaurosis without any associated headache, scalp tenderness, or jaw claudication.

**Figure 1:**
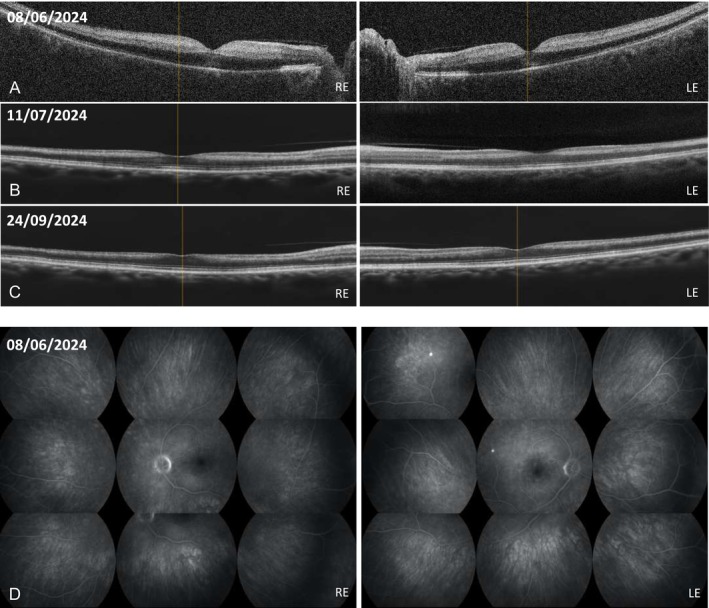
OCT scans: retinal edema with ischemic hyperreflectivity of the internal neuroretinal layers (A) right after the onset of visual loss. At (B) one month and (C) three months after initial onset, the OCT scan revealed loss/thinning of inner retinal layers with minimal foveal contour. The outer retinal layers remained intact. (D) Wide‐field fluorescein angiography: markedly delayed arterial filling and diffuse retinal hypoperfusion were present right after the onset of visual loss, with early optic disc hyperfluorescence indicating dye leakage; no emboli or vascular tortuosity was observed. LE, left eye; OCT, optical coherence tomography; RE, right eye.

On ophthalmologic evaluation, the patient exhibited markedly reduced visual acuity in both eyes accompanied by a relative afferent pupillary defect. Fundus examination revealed pale optic discs with distinct central cherry‐red spots. Optical coherence tomography further demonstrated marked hyperreflectivity and edema of the inner retinal layers (Figure 1A–C), and fluorescein angiography confirmed delayed retinal arteriolar filling (Figure 1D), findings that were consistent with a diagnosis of bilateral CRAO.

Laboratory studies revealed significantly elevated markers of inflammation, including a C‐reactive protein level of 166 mg/L, a fibrinogen level of 612 mg/dL, and an erythrocyte sedimentation rate of 43 mm/h. Additionally, antimyeloperoxidase (anti‐MPO) antibodies were positive at a titer of 134 IU/L, whereas the peripheral eosinophil count remained within the normal range, likely reflecting the ongoing effects of benralizumab. A comprehensive diagnostic evaluation, which included transthoracic echocardiography, carotid Doppler ultrasound, a computed tomography scan of the chest, and a thrombophilia screening, excluded embolic, cardiac, and atherosclerotic causes. In light of the ocular complications, benralizumab therapy was promptly discontinued.

## Differential diagnosis

The differential diagnosis included the following:

- embolic or thrombotic CRAO due to atherosclerotic disease
- cardioembolic events
- other systemic vasculitis, such as granulomatosis with polyangiitis

The absence of embolic sources and the presence of anti‐MPO antibodies, combined with the patient's clinical history, supported the diagnosis of EGPA with predominant vasculitic complications.

## Management and outcome

Based on the severity of the presentation, an aggressive immunosuppressive regimen was promptly initiated. For induction, high‐dose glucocorticoid pulses were administered at 1,000 mg intravenously daily for four days, followed by tapering according to the PEXIVAS guidelines.^2^ In addition, the patient was treated with a combination protocol of rituximab and cyclophosphamide, following the “Kidney Disease: Improving Global Outcomes” organization (KDIGO) recommendations,^3^ with rituximab given at 375 mg/m^2^ weekly for four weeks alongside cyclophosphamide at 15 mg/kg on weeks zero and two. This combined approach was chosen to target both the ANCA‐mediated vascular injury and the underlying autoimmune activity. Despite initial clinical improvement, the patient experienced a relapse characterized by constitutional symptoms and a rise in anti‐MPO titers. In response, an additional single pulse of glucocorticoids was administered, and the patient received two further monthly intravenous doses of cyclophosphamide, achieving a cumulative dose of 4 g. Following these adjustments, systemic markers of inflammation normalized within six months (by December 2024); however, visual acuity remained severely compromised, with the best‐recorded measurements being finger counting at 1 meter in the right eye and a visual acuity of 2/10 in the left eye. For maintenance therapy, rituximab was continued according to the Mainritsan protocol,^4^ aiming to sustain immunosuppression and prevent further relapses of systemic vasculitis.

## Discussion

EGPA typically presents with a prodromal phase of asthma and rhinosinusitis, progressing to vasculitic manifestations often associated with ANCA positivity. In this case, benralizumab effectively suppressed eosinophilia but failed to prevent ANCA‐mediated vascular injury. This suggests that IL‐5/IL‐5 receptor blockade may not be sufficient to control vasculitic activity, reflecting distinct pathogenic pathways for eosinophilic and ANCA‐driven damage. Early, aggressive immunosuppression remains essential to prevent irreversible organ involvement, even in the era of targeted biologic therapies.^5^

## Conclusions

This case represents the first reported occurrence of EGPA presenting with a vasculitic complication (CRAO) during ongoing anti–IL‐5 receptor therapy. Prompt and intensive immunosuppression with a combination of rituximab and cyclophosphamide led to partial, albeit limited, improvement in visual acuity.

## AUTHOR CONTRIBUTIONS

All authors contributed to at least one of the following manuscript preparation roles: conceptualization AND/OR methodology, software, investigation, formal analysis, data curation, visualization, and validation AND drafting or reviewing/editing the final draft. As corresponding author, Dr Quartuccio confirms that all authors have provided the final approval of the version to be published and takes responsibility for the affirmations regarding article submission (eg, not under consideration by another journal), the integrity of the data presented, and the statements regarding compliance with institutional review board/Declaration of Helsinki requirements.

REFERENCES1

André
R
, 
Cottin
V
, 
Saraux
JL
, et al; French Vasculitis Study Group (FVSG). Central nervous system involvement in eosinophilic granulomatosis with polyangiitis (Churg‐Strauss): report of 26 patients and review of the literature. Autoimmun Rev
2017;16(9):963–969.28709761
10.1016/j.autrev.2017.07.0072

Walsh
M
, 
Merkel
PA
, 
Peh
CA
, et al; PEXIVAS Investigators. Plasma exchange and glucocorticoids in severe ANCA‐associated vasculitis. N Engl J Med
2020;382(7):622–631.32053298
10.1056/NEJMoa1803537PMC73257263

Kidney
Disease
: Improving Global Outcomes (KDIGO) ANCA Vasculitis Work Group. KDIGO 2024 clinical practice guideline for the management of antineutrophil cytoplasmic antibody (ANCA)‐associated vasculitis. Kidney Int
2024;105(3 suppl):S71–S116.38388102
10.1016/j.kint.2023.10.0084

Tanna
A
, 
Pusey
C
. Rituximab for maintenance of remission in AAV. Nat Rev Nephrol
2015;11(3):131–132.25599619
10.1038/nrneph.2014.2545

Hellmich
B
, 
Sanchez‐Alamo
B
, 
Schirmer
JH
, et al. EULAR recommendations for the management of ANCA‐associated vasculitis: 2022 update. Ann Rheum Dis
2024;83(1):30–47.36927642
10.1136/ard-2022-223764
